# Ozone-triggered surface uptake and stress volatile emissions in *Nicotiana tabacum* ‘Wisconsin’

**DOI:** 10.1093/jxb/erx431

**Published:** 2017-12-30

**Authors:** Arooran Kanagendran, Leila Pazouki, Shuai Li, Bin Liu, Astrid Kännaste, Ülo Niinemets

**Affiliations:** 1 Institute of Agricultural and Environmental Sciences, Estonian University of Life Sciences, Tartu, Estonia; 2 Department of Biology, University of Louisville, Louisville, KY, USA; 3 Estonian Academy of Sciences, Tallinn, Estonia

**Keywords:** Acute ozone stress, *de novo* emission, elicitation rate, glandular trichome, LOX volatiles, monoterpenes, non-stomatal ozone deposition, sesquiterpenes, solvent extract, stomatal ozone uptake, trichome permeability

## Abstract

Ozone is a strong oxidant and a key stress elicitor. The immediate and longer term impacts of ozone are poorly understood in species with emission of both *de novo* synthesized and stored volatiles, such a tobacco (*Nicotiana tabacum*), which has terpene-containing glandular trichomes on the leaf surface. In this study, we exposed *N. tabacum* ‘Wisconsin’ leaves to acute ozone doses of 0 (control), 400, 600, 800, and 1000 ppb for 30 min and studied the effects of ozone exposure on ozone uptake, gas-exchange characteristics, and emissions of lipoxygenase pathway volatiles, monoterpenes, and sesquiterpenes. Foliage emissions of lipoxygenase pathway volatiles were quantitatively related to the severity of ozone exposure, but the stress dose *vs*. emission relationship was weaker for terpenoids. Analysis of leaf terpene content and composition indicated that several monoterpenes and sesquiterpenes were not stored in leaves and were synthesized *de novo* upon ozone exposure. The highest degree of elicitation for each compound was observed immediately after ozone treatment and it declined considerably during recovery. Leaf ozone uptake was dominated by non-stomatal deposition, and the emissions of total lipoxygenase pathway volatiles and mono- and sesquiterpenes were positively correlated with non-stomatal ozone deposition. Overall, this study demonstrates remarkably high ozone resistance of the studied tobacco cultivar and indicates that ozone’s effects on volatile emissions primarily reflect modifications in the release of stored volatiles and reaction of ozone with the leaf surface structure.

## Teaser

Ozone-driven isoprenoid emissions scaled with surface ozone uptake, indicating that isoprenoids were released from trichomes. Greater non-stomatal ozone uptake due to ozone detoxification on the leaf surface underlies greater ozone tolerance.

## Introduction

Ozone (O_3_) is a phytotoxic gas that leads to major losses of vegetation productivity worldwide (Fares *et al.*, 2010*b*). In the troposphere, ozone formation is controlled by concentrations of NO_2_ and reactive volatile organic compounds in the presence of sunlight (Fowler *et al.*, 2009; Karnosky *et al.*, 2005). Currently, tropospheric ozone concentration over most of the terrestrial surface is between 20 and 45 ppb (Sicard *et al.*, 2017), but in local pollution hotspots, much higher concentrations can be observed, e.g. in major industrial cities in China, the ozone concentration is *ca* 70 ppb (Yuan *et al.*, 2015) and in industrial cities in India, it is *ca* 50–60 ppb (Sharma *et al.*, 2013). However, even the current average levels of tropospheric ozone are capable of having a detrimental effect on certain sensitive crop plants, such as wheat, maize, and soybean (Rai and Agrawal, 2014).

Plants act as a sink for ozone in two ways: stomatal uptake and non-stomatal deposition (Altimir *et al.*, 2006; Fares *et al.*, 2010*b*). During stomatal uptake, ozone diffuses through the stomata into the leaf intercellular air spaces along the ozone gradient from the ambient air (Laisk *et al.*, 1989). Stomatal uptake is the primary route for ozone into the leaf mesophyll cells (Fares *et al.*, 2008). Once taken up, ozone, as a strong oxidant, can result in major oxidative stress (Schwanz *et al.*, 1996; Calfapietra *et al.*, 2013).

There is still a large uncertainty over how plant stress severity scales with ozone uptake. In particular, counterintuitively, less physiological damage has been observed in species with greater ozone uptake, especially in species with greater volatile emissions (Fares *et al.*, 2010*b*; Kanagendran *et al.*, 2018). Yet, many model species also have significant volatile-containing surface structures, in particular glandular trichomes, and it is currently unclear whether the greater ozone tolerance of species with greater ozone uptake is associated with ozone detoxification inside the leaves or on the leaf surface (Jardine *et al.*, 2012; Jud *et al.*, 2016).

*Nicotiana tabacum* L. is an annual herb that has terpene-filled glandular trichomes on the leaf surface and has therefore been used as a model species for ozone–plant surface reactions (Jud *et al.*, 2016). There have been several studies looking at the physiological impact of ozone on *N. tabacum* (Beauchamp *et al.*, 2005; Janzik *et al.*, 2005; Samuel *et al.*, 2005; Eltayeb *et al.*, 2006; Heidenreich *et al.*, 2006; Mancini *et al.*, 2006; Cheng and Sun, 2013; Pasqualini *et al.*, 2009; Silva *et al.*, 2012), but the majority of studies have mainly focused on short-term immediate stress responses rather than considered the full response from exposure through the recovery period. There is also a lack of knowledge of how the emissions of the lipoxygenase pathway (LOX) volatiles (also called green leaf volatiles), mono- and sesquiterpenes are regulated upon acute ozone doses from exposure through the recovery period and their relationship with foliage surface ozone uptake and elicitation rate.

There is a significant variability in ozone sensitivity among *N. tabacum* cultivars but *N. tabacum* ‘Bel W3’ is more sensitive to ozone than many other crop plants and therefore often used as an ozone bioindicator (Schraudner *et al.*, 1998; Krupa *et al.*, 2001). In particular, the study of Heiden *et al.* (1999) compared ozone-induced volatile emissions between the ozone-resistant cultivar ‘Bel B’ and ozone-sensitive cultivar ‘Bel W3’ and found that the elicitation of LOX volatiles and volatile isoprenoid release depended strongly on both the duration of ozone exposure and the cultivar. However, it is unclear how more resistant cultivars with potentially a greater share of surface deposition flux respond to acute ozone exposure and how immediate stress responses and gene-expression-level elicitation responses correlate with the ozone dose.

In particular, elicitation of *de novo* volatile emissions by activation of a gene-expression-level response upon ozone exposure is expected to increase through the recovery phase, while the release of volatiles from the leaf surface is expected to decrease during recovery. Thus, time kinetics of volatile responses upon ozone exposure can provide important insight into the development of oxidative stress vis-à-vis defence responses and explain non-intuitive ozone dose *vs*. physiological plant responses in different tobacco cultivars.

In this study, we used short-term acute (400–1000 ppb) ozone exposures and studied the changes in photosynthetic characteristics, emissions of LOX volatiles and mono- and sesquiterpenes, stomatal ozone uptake rates, non-stomatal ozone deposition rates, and stress-dependent elicitation rates of each volatile through the recovery phase in the ozone-resistant *N. tabacum* ‘Wisconsin’. In addition, terpenoid content and composition in untreated leaves were also studied to confirm the capacity for terpenoid storage by leaves and allow separation of stored and *de novo* synthesized terpene sources. The objectives of this study were to (a) assess the relationships between ozone concentration and changes in foliage photosynthetic characteristics and emissions of LOX volatiles and mono- and sesquiterpenes from ozone exposure on through the recovery phase, and (b) distribute leaf ozone uptake between stomatal ozone uptake and surface deposition and relate the different components of ozone flux to the degree of elicitation of different volatiles. Different stressed-induced mechanisms varying from gene expression to allocation of primary intermediates can be responsible for stress-dependent changes in volatile emission.

We hypothesized that acute ozone exposure to foliage of this *N. tabacum* ozone-resistant cultivar will lead to (a) moderate reductions in gas exchange characteristics, (b) greater non-stomatal deposition rate of ozone than stomatal uptake rate, (c) alteration in LOX volatile and mono- and sesquiterpene emission rates primarily due to changes in the constitutive emissions, most likely associated with changes in trichome surface permeability, and (d) changes in stress-dependent elicitation rates of LOX volatile, mono- and sesquiterpene emissions.

## Materials and methods

### Plant material and growth conditions

Tobacco (*N. tabacum* ‘Wisconsin’) seeds were sown in 1-litre plastic pots filled with commercial potting soil (Biolan Oy, Kekkilä group, Finland). Four weeks after germination, seedlings were transplanted into 3-litre plastic pots filled with the same potting soil. The plants were maintained for the whole experimental period under a light intensity of 400–500 µmol m^−2^ s^−1^ provided by metal halide lamps (HPI-T Plus 400 W, Philips) with a 12 h photoperiod, relative humidity of 60% and day/night temperature of 24/18 °C. Plants were watered daily to soil field capacity. In addition to the nutrients provided by the soil, a liquid fertilizer (Baltic Agro, Lithuania; NPK content ratio: 5:5:6; and micronutrients B (0.01%), Cu (0.03%), Fe (0.06%), Mn (0.028%), and Zn (0.007%) was applied for optimum growth conditions. Once a week, 70 ml of diluted liquid fertilizer (*ca* 0.5 % solution) was applied to each plant until the end of the study.

### Experimental design and data collection

The experiment was conducted with the leaves of 10- to 12-week-old plants. Fully mature similar-sized leaves from similar canopy positions and a similar leaf order were used for the experiment. For each of the five ozone exposure concentrations, five arbitrarily selected leaves from a single plant were used to measure gas exchange and volatile emission rates at five recovery time points, and stomatal ozone uptake and non-stomatal ozone deposition during ozone exposures. Each leaf from each plant was separately treated with the same ozone concentration and sampled at a single recovery time point. All exposures were replicated with three individual plants including control measurements. Therefore, in total, 75 leaves (5 recovery time points × 3 plants for each exposure × 5 exposures including 0, 400, 600, 800, and 1000 ppm ozone) from 15 plants were used. In addition, there were six untreated leaves from six plants used for the estimation of contents and composition of foliage terpenoids by solvent extract analysis. This experimental design does not allow for direct assessment of leaf-to-leaf variability in emission responses, but treated leaves were measured only once to avoid possible leaf damage and release of surface volatiles when the leaves had to be removed, inserted and sealed in the leaf chamber repeatedly (see Niinemets *et al.* (2011) for discussion of the caveats of plant volatile measurements).

### Experimental set-up and ozone exposure

A custom-made cylindrical double-walled glass chamber (1.2 litres) with stainless steel bottom was used for ozone treatments, gas exchange measurements and volatile sampling. As the leaf was the experimental unit in this study, individual leaves were placed in the 1.2-litre glass chamber for ozone treatment. The chamber temperature was maintained constant at 25 ºC by circulating thermostatted water between the double walls of the glass chamber. Light was provided by four 50 W halogen lamps. A thermistor (NTC thermistor, model ACC-001, RTI Electronics, Inc., Anaheim, CA, USA) was used to monitor the air temperature inside the chamber, and leaf temperature was measured by a thermocouple attached to the lower side of the leaf surface. Ambient air was filtered through activated charcoal and supplied to the chamber in the form of pure and ozone-free air. A fan inserted inside the chamber provided constant high turbulence air mixing. The chamber ports could be switched between reference and measurement modes in order to take gas-exchange measurements (see Copolovici and Niinemets (2010) for further details of the glass chamber set-up). CO_2_ and H_2_O concentrations of the chamber inlet and outlet were measured with an infra-red dual-channel gas analyser operated in differential mode (CIRAS II, PP Systems, Amesbury, MA, USA).

Ozone was generated with a Certizon C100 ozoniser (Erwin Sander Elektroapparatenbau GmbH, Germany). Arbitrarily selected mature leaves were exposed to 400, 600, 800, and 1000 ppb ozone for 30 min, and untreated leaves were used as control. The ozone concentration in the inlet and outlet of the chamber was monitored using a UV photometric ozone detector (Model 49i, Thermo Fisher Scientific, Franklin, MA, USA).

### Gas exchange measurements, volatile sampling, and gas chromatography–mass spectrometry analyses

After leaf enclosure, standard environmental conditions were established: light intensity at leaf surface of 700 μmol m^−2^ s^−1^, leaf temperature of 25 ^o^C, ambient CO_2_ concentration of 380–400 μmol mol^−1^, and relative air humidity of 50–60%. Once the foliage gas-exchange rates had reached a steady state, usually after *ca* 15 min of leaf enclosure, foliage gas exchange rates were recorded and volatiles were collected (control). Then the leaf was exposed to ozone for 30 min, and the measurements were repeated at 0.5, 3, 10, 24, and 48 h after the exposure. Net assimilation rate (*A*), and stomatal conductance to water vapour (*g*_s_) were calculated according to von Caemmerer and Farquhar (1981).

Volatile samples were collected onto multi-bed stainless steel adsorbent cartridges connected to a portable suction pump, 210–1003 MTX (SKC Inc., Houston, TX, USA) providing a constant suction rate of 200 ml min^−1^. A detailed description of the method and adsorbent cartridges can be found in Kännaste *et al.* (2014) and Niinemets *et al.* (2010). An air sample was taken before the leaf enclosure to estimate the chamber volatile background without the leaf. Adsorbent cartridges were analysed with a combined Shimadzu TD20 automated cartridge desorber and Shimadzu 2010 Plus gas chromatography–mass spectrometry (GC-MS) system (Shimadzu Corporation, Kyoto, Japan) for LOX volatiles, monoterpenes, and sesquiterpenes (Kännaste *et al.* (2014) and Toome *et al.* (2010) for further details about GC-MS analysis and compound identification), and the emission rates were calculated according to Niinemets *et al.* (2011).

### Quantitative estimation of foliage terpene contents

Terpene content was quantitatively estimated from untreated leaves. For the chemical analyses of foliage terpene content, six fresh leaf samples (120 ± 30 mg) were randomly chosen from individual plants and weighed into a 2 ml tube with 2.8 mm stainless steel beads (Bertin Technologies, Aix-en-Provence, France). Tubes with fresh samples were instantly frozen in liquid N_2_ and 1.5 ml GC-grade hexane (Sigma-Aldrich, St Louis, MO, USA) was added into each tube. The mixture was then immediately homogenized with a homogenizer (Precellys 24, Bertin Technologies) at 2400 *g* for 30 s at 25 °C. The homogenized plant material was incubated in a Thermo-Shaker (TS-100C, Biosan, Riga, Latvia) for 3 h at 1000 rpm at 25 °C. After incubation, samples were centrifuged (Universal 320R, Hettich, Tuttlingen, Germany) for 30 min at 13 200 *g* at 25 °C. An aliquot of 1 ml supernatant was pipetted into a 2 ml GC-MS glass vial (Sigma-Aldrich) for GC-MS analysis. A detailed description of GC-MS analysis and quantitative estimation of foliage terpene contents can be found in Kännaste *et al.* (2013).

### Chlorophyll florescence measurements

Dark-adapted chlorophyll fluorescence yield (10 min darkening, *F*_v_*/F*_m_) of each ozone-treated leaf was estimated with a PAM fluorimeter (Walz IMAG-MIN/B, Walz GmbH, Effeltrich, Germany) after gas exchange measurements and volatile sampling were completed. A saturating pulse intensity of 7000 µmol m^−2^ s^−1^ was used.

### Estimation of ozone uptake by individual leaves

Average stomatal ozone uptake rate and non-stomatal ozone deposition rate for the exposure period by each individual leaf at each dose of ozone was calculated from measurements of chamber incoming and outgoing ozone concentrations. First, total ozone uptake flux (Φ_O3,Tot_) was calculated as the product of chamber flow rate and difference in ozone concentrations between chamber inlets and outlets.

Average stomatal ozone uptake rate (Φ_O3,S_) was calculated as the product of average chamber O_3_ concentration and average stomatal conductance for ozone (*g*_O3_). The latter was estimated as the 30-min average stomatal conductance to water vapour divided by the ratio of the binary diffusion coefficients for ozone and water vapour (2.03) assuming that the intercellular ozone concentration was zero (Laisk *et al.*, 1989). This assumption might overestimate ozone uptake as ozone concentrations in the leaf interior can reach small non-zero values (Moldau and Bichele, 2002). The ratio of water vapour and ozone diffusivities was calculated using an ozone diffusion coefficient in air of 1.267 × 10^−5^ m^2^ s^−1^ at 22.84 ºC (Ivanov *et al.*, 2007) and a water vapour diffusion coefficient in air of 2.569 × 10^−5^ m^2^ s^−1^ at the same temperature according to the theory of Chapman and Enskog (Li *et al.*, 2017; Niinemets and Reichstein, 2003). In fact, as the ratio of the collision integrals for both water vapour and O_3_ changes little with temperature (calculated according to Tucker and Nelken, 1982), temperature effects on this ratio were negligible (the change in the ratio was less than 0.1% between 25 ºC used for leaf measurements and 22.84 ºC).

Cuticular ozone uptake into the leaf interior was excluded because compared with stomata, the cuticle is highly impermeable to ozone (Kerstiens and Lendzian, 1989). Ozone deposition flux was estimated as the difference between Φ_O3,Tot_ and Φ_O3,S_. A detailed description of quantifying stomatal ozone flux and non-stomatal ozone deposition can be found in Winner (1994). Stomatal ozone uptake rate and non-stomatal ozone deposition rate were calculated for each ozone exposure.

### Data analyses

Measurements were replicated three times with each ozone concentration. A generalized linear model (GLM) with maximum likelihood model fitting was used to test for individual and interactive effects of ozone and the time of recovery on the emissions of total LOX volatiles, total monoterpenes, total sesquiterpenes, individual mono- and sesquiterpenes, and elicitation rate of each compound. The elicitation rates of log-transformed LOX volatiles, and mono- and sesquiterpene emission rates were calculated using the data fitted to a model selected based on the emission pattern of each compound (Beauchamp *et al.*, 2005). Here we used the sigmoidal model in the form:

$$y=y_{0}+\frac{\alpha }{1+e^{-\left(\frac{x-x_{0}}{b}\right)}}$$

In this case, the elicitation rate of each compound emission was calculated at recovery times 0.5, 10, and 48 h, compared with the emission of corresponding compounds from control leaves. Here, *y*_0_ is the initial level of emission (emission of control) at time *x*_0_ (0 h for control emission), *a* is the saturation level of emission (emission of treatment sample at each recovery point), *x* is the recovery time (such as 0.5, 10, and 48 h), and (1/*b*) is the elicitation rate. The elicitation rate of log-transformed emission data at each recovery point was calculated for each replicate and then the mean (±SE) elicitation rate was estimated.

Emission data were log-transformed after checking for normality of distribution of data and homogeneity of variances, in order to minimize the inequality of variances for Wald’s chi-square test in the GLM. Correlation analyses of net assimilation rate, intercellular CO_2_ concentration, and stomatal conductance to water vapour *vs*. ozone doses at different times through recovery were carried out. In addition, correlations of total LOX volatile, total monoterpene, and total sesquiterpene emission rates with stomatal ozone uptake rate and non-stomatal ozone deposition rate at different recovery periods were also examined by non-parametric Spearman correlations.

Elicitation rate calculation was carried out using SigmaPlot v. 12.5 (Systat Software Inc., San Jose, CA, USA). All other statistical analyses were conducted using SPSS Statistics v. 24 (IBM Corp., Armonk, NY, USA). All statistical effects were considered significant at *P*<0.05.

## Results

### Effects of ozone stress on foliage photosynthetic characteristics

**Fig. 1.**
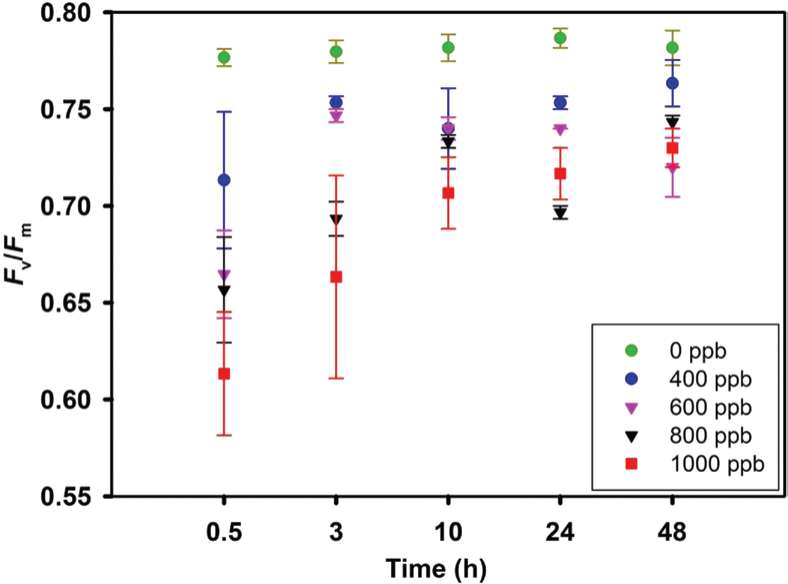
Dark-adapted (10-min darkening) average (±SE) maximum fluorescence yield of photosystem II (*F*_v_/*F*_m_) of mature leaves of 10- to 12-week-old *Nicotiana tabacum* ‘Wisconsin’ at 0.5, 3, 10, 24, and 48 h of recovery following the exposure to ozone concentrations of 0, 400, 600, 800, and 1000 ppb for 30 min in a custom-made cylindrical double-walled glass chamber at 25 °C. All measurements were replicated at least three times. (This figure is available in color at *JXB* online.)

The maximum dark-adapted photosystem II (PSII) quantum yield (*F*_v_*/F*_m_) significantly decreased in leaves exposed to 600–1000 ppb ozone; however, 400 ppb exposure of ozone caused only a marginal decline in *F*_v_*/F*_m_ (Fig. 1). *F*_v_*/F*_m_ at 0.5 h after the exposure was negatively correlated with ozone concentration (*r*^2^=0.73, *P*<0.001, data not shown). For leaves exposed to 400 ppb, *F*_v_*/F*_m_ had recovered almost fully at 48 h after exposure, but the recovery was only partial for leaves exposed to 600–1000 ppb ozone (Fig. 1).

**Fig. 2.**
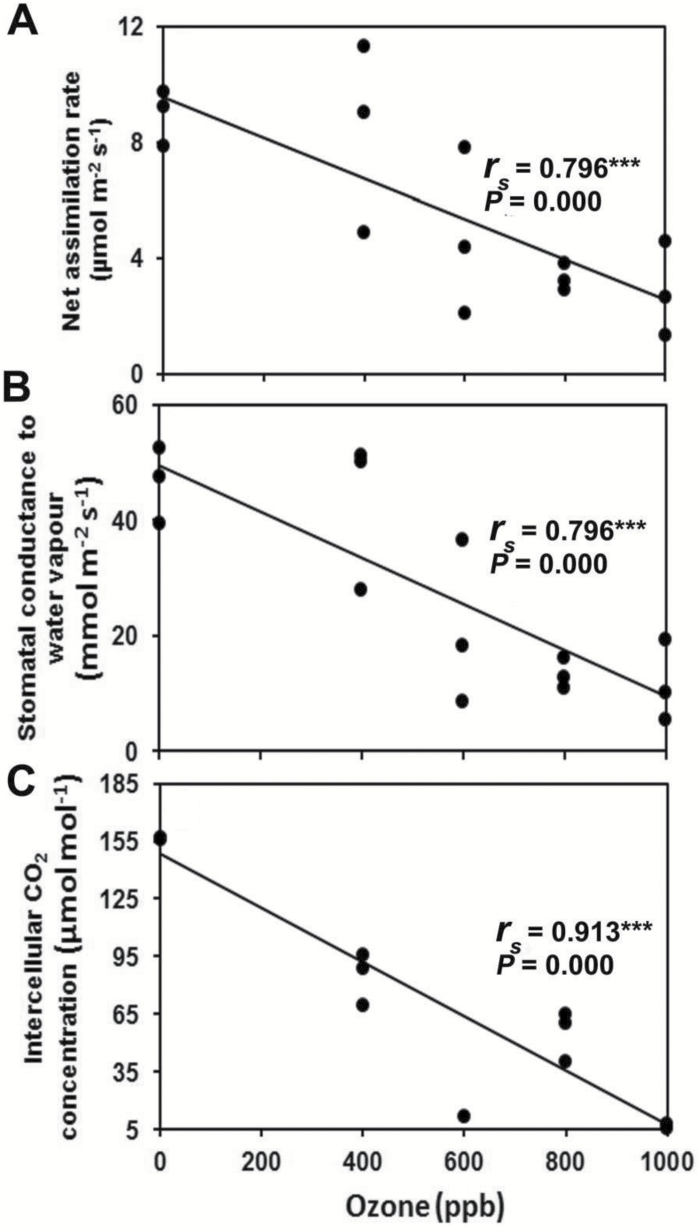
Changes in leaf net assimilation rate (A), stomatal conductance to water vapour (B), and intercellular CO_2_ concentration (C) of mature leaves of 10- to 12-week-old *N. tabacum* ‘Wisconsin’ in relation to ozone concentration during exposure. The measurements were taken at 0.5 h after ozone exposure at 0, 400, 600, 800, and 1000 ppb for 30 min in a custom-made cylindrical double-walled glass chamber at 25 °C. All measurements were replicated at least three times. Gas exchange measurements were conducted at leaf temperature of 25 °C, quantum flux density of 700 μmol m^−2^ s^−1^ at leaf surface, ambient CO_2_ concentration of 380–400 μmol mol^−1^, and relative air humidity of 50–60%. Each data point corresponds to an individual replicate measurement. Data were fitted by Spearman correlations. Statistical significance of regressions is indicated as ***P<0.001.

Exposure of ozone led to a statistically significant reduction in net assimilation rate (*A*; Fig. 2A), stomatal conductance to water vapour (*g*_*s*_; Fig. 2B), and intercellular CO_2_ concentration (*C*_i_; Fig. 2C) for all exposure concentrations. However, net assimilation rate, stomatal conductance to water vapour, and intercellular CO_2_ concentration completely recovered after 48 h of recovery for all treatments (see Supplementary Fig. S1 at *JXB* online).

### Emission of foliar LOX volatiles in relation to ozone dose and time of recovery

**Fig. 3.**
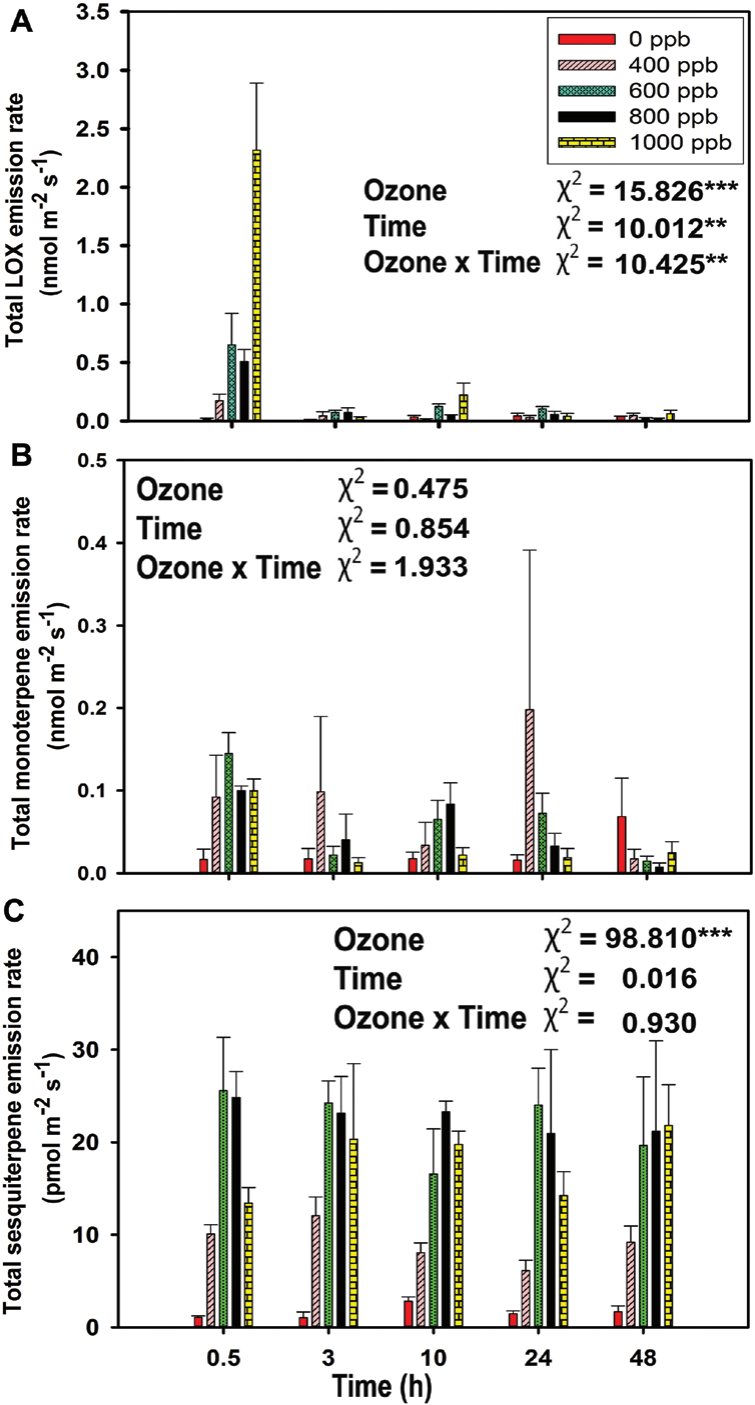
Average (+SE) emission rates of total lipoxygenase pathway volatiles (LOX volatiles or green leaf volatiles, A), total monoterpenes (B), and total sesquiterpenes (C) in mature leaves of 10- to 12-week-old *N. tabacum* ‘Wisconsin’ at 0.5, 3, 10, 24, and 48 h of recovery after ozone exposure at 0, 400, 600, 800, and 1000 ppb for 30 min in a custom-made cylindrical double-walled glass chamber at 25 °C. Data are averages of three independent replicates. Individual effects of ozone, recovery time (Time), and their interactions on emission rates were tested by GLM with maximum likelihood model fitting. Wald’s chi-square (χ^2^) test statistics and statistical significance are indicated as **P<0.01, ***P<0.001. (This figure is available in color at *JXB* online.)

**Fig. 4.**
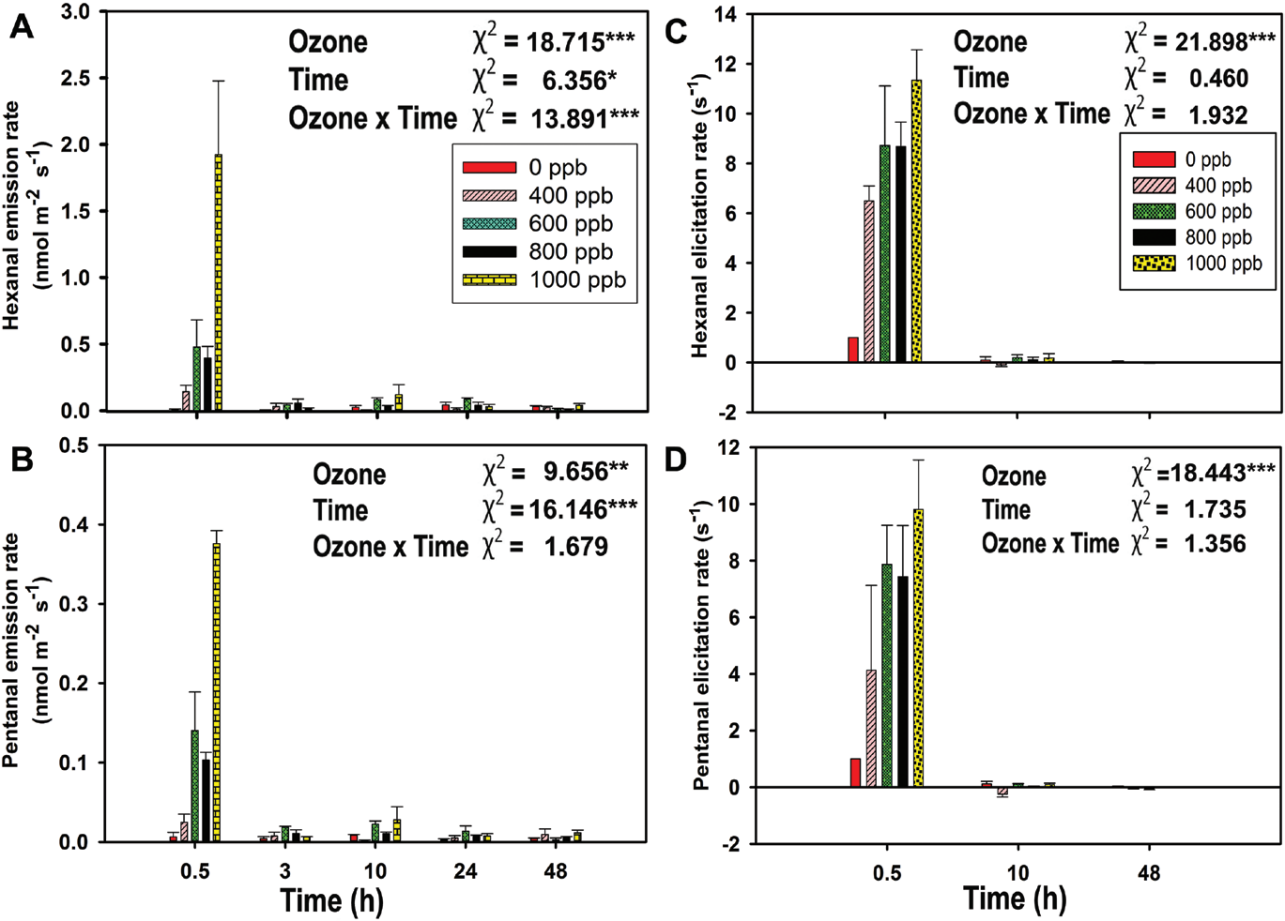
Average (+SE) emission rates of the individual lipoxygenase pathway volatiles (LOX volatiles) hexanal (A) and pentanal (B) and average (+SE) elicitation rate of hexanal (C) and pentanal (D) in mature leaves of 10- to 12-week-old *N. tabacum* ‘Wisconsin’ at different recovery times (0.5, 3, 10, 24, and 48 h for emission rate and 0.5, 10, and 48 h for elicitation rate) after ozone exposure at 0, 400, 600, 800, and 1000 ppb for 30 min in a custom-made cylindrical double-walled glass chamber at 25 °C. The elicitation rate of emissions is defined as the rate of change of emission (positive values indicate an increase of the rate and negative values a decrease of the rate). Data are averages of three independent replicates. Individual effects of ozone, recovery time (Time), and their interactions on emission rates were tested by GLM with maximum likelihood model fitting. Wald’s chi-square (χ^2^) test statistics and statistical significance are indicated as *P<0.05, **P<0.01, ***P<0.001. (This figure is available in color at *JXB* online.)

In non-treated leaves, LOX volatiles were emitted at a very low level (mean±SE=0.03 ± 0.01 nmol m^−2^ s^−1^). Ozone treatment strongly enhanced the emission of LOX volatiles after ozone exposure (Figs 3A and 4A, B). The total emission of LOX volatiles was highest immediately after ozone exposure and decayed over the recovery phase to the pre-stress level (Fig. 3A, *P*<0.001 for ozone treatments and *P*<0.01 for the recovery time). The statistically significant ozone concentration effect reflected the highest rate of total LOX volatile emission at 1000 ppb ozone, a *ca* 160-fold higher rate compared with the control, followed by the intermediate rates at 600 and 800 ppb, and minor enhancement at 400 ppb (Fig. 3A).

The ozone-elicited LOX volatile emissions were dominated by the saturated aldehydes hexanal and pentanal throughout the time of recovery, and as for total LOX volatile emissions, the emissions were greatest at 0.5 h after the ozone exposure. In this case, ozone and recovery time had a statistically significant impact on LOX volatile emission rates (Figs 3A and 4A, B). In this study, emission of the classic LOX volatiles (*Z*)-3-hexenol and (*E*)-2-hexenal was observed at trace levels only at 1000 ppb ozone dose (data not shown).

### Emission responses of foliar mono- and sesquiterpenes in relation to ozone exposure and time of recovery

**Fig. 5.**
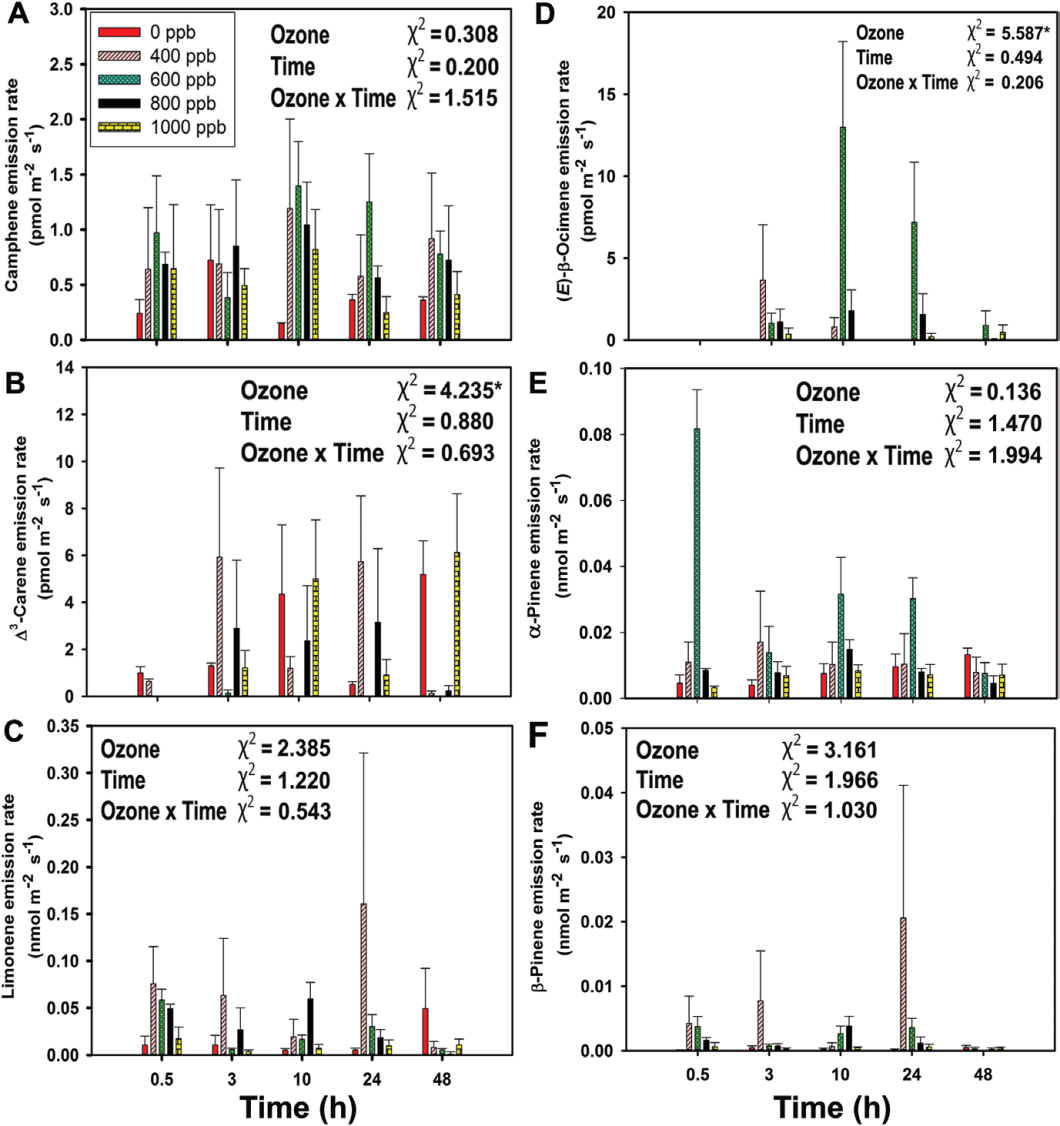
Average (+SE) emission rates of key monoterpenes (A–F) from mature leaves of 10- to 12-week-old *N. tabacum* ‘Wisconsin’ at 0.5, 3, 10, 24, and 48 h of recovery after ozone exposure at 0, 400, 600, 800, and 1000 ppb for 30 min in a custom-made cylindrical double-walled glass chamber at 25 °C. Individual effects of ozone, recovery time (Time), and their interactions on emission rates were tested by GLM with maximum likelihood model fitting. Wald’s chi-square (χ^2^) test statistics and statistical significance are indicated as *P<0.05. (This figure is available in color at *JXB* online.)

**Fig. 6.**
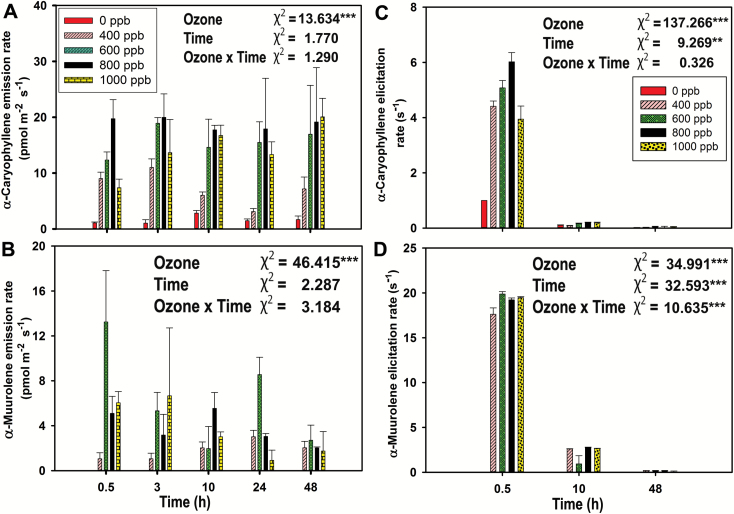
Average (+SE) emission rates of α-caryophyllene (A) and α-muurolene (B) and average (+SE) elicitation rate of α-caryophyllene (C) and α-muurolene (D) in mature leaves of 10- to 12-week-old *N. tabacum* ‘Wisconsin’ at different recovery times (0.5, 3, 10, 24, and 48 h for emission rate and 0.5, 10, and 48 h for elicitation rate) after ozone exposure at 0, 400, 600, 800, and 1000 ppb for 30 min in a custom-made cylindrical double-walled glass chamber at 25 °C. The elicitation rate of emissions is defined as the rate of change of emission (positive values indicate an increase of the rate and negative values a decrease of the rate). Data are averages of three independent replicates. Individual effects of ozone, recovery time (Time), and their interactions on emission rates were tested by GLM with maximum likelihood model fitting. Wald’s chi-square (χ^2^) test statistics and statistical significance are indicated as **P<0.01, ***P<0.001. (This figure is available in color at *JXB* online.)

Non-treated leaves of *N. tabacum* were low-level emitters of monoterpenes (mean±SE rate of emission of 0.02 ± 0.01 nmol m^−2^ s^−1^) and sesquiterpenes (1.66 ± 0.25 pmol m^−2^ s^−1^) (Fig. 3B, C). In control leaves, the blend of emitted monoterpenes was dominated by limonene, followed by α-pinene, β-pinene, Δ^3^-carene, and camphene (Fig. 5), whereas α-caryophyllene was observed as the main sesquiterpene (Fig. 6A). In addition, the monoterpene (*E*)-β-ocimene (Fig. 5D) and the sesquiterpene α-muurolene were emitted upon ozone exposure (Fig. 6B).

The effect of ozone concentration on monoterpene emissions was non-linear with the highest emissions initially observed for 600 ppb exposure at 0.5 h since the exposure (Fig. 3B). The concentration effect was more clearcut for sesquiterpene emissions to 600 ppb of exposure, and then the emission decreased with increasing ozone concentration (Fig. 3C). Monoterpene emissions of ozone-treated leaves remained similar through the recovery, except at the end of the recovery at 48 h when the emissions of treated leaves had decreased below the values observed for control leaves (Fig. 3B). In contrast, no such decline was observed for sesquiterpenes (Fig. 3C).

The time courses of individual monoterpene emissions broadly reflected the variation in total monoterpene emissions, except for Δ^3^-carene, for which ozone concentration and time had a minor impact on the emissions (Fig. 5B). In addition, there were some differences in reaching peak emission and modified emission patterns through recovery for different monoterpenes. In particular, β-pinene and limonene emissions tended to peak in the leaves exposed to 400 ppb ozone at 24 h after exposure (Fig. 5C, F), but α-pinene emissions peaked at 600 ppb ozone exposure and at 0.5 h since the exposure (Fig. 5E). The emission of the characteristic stress-induced monoterpene, (*E*)-β-ocimene, was first observed after 3 h since ozone treatment, and the emission peaked for 600 ppb ozone at 10 h since the exposure (Fig. 5D).

In the case of sesquiterpenes, ozone concentration and time of recovery affected α-caryophyllene emissions similarly to total sesquiterpene emissions (Figs 3C and 6A). In contrast, there were no emission responses of α-muurolene observed from control leaves and the highest α-muurolene emissions were observed for 600 ppb ozone treatment at 0.5 h since the exposure, and the emissions decreased slightly through the recovery phase (Fig. 6B). Furthermore, we have observed γ-cadinene and γ-muurolene at trace levels in response to 600–1000 ppb ozone exposures (data not shown).

### Quantitative estimation of terpene content in *Nicotiana tabacum* leaves

Four monoterpenes, Δ^3^-carene, limonene, α-pinene, and β-pinene, were stored in the tissues of untreated leaves (Table 1). Δ^3^-Carene was the most abundant monoterpene observed in the leaf tissues followed by α-pinene, limonene, and β-pinene. The sesquiterpenes α-caryophyllene and α-muurolene were not observed at a detectable level in leaf tissues (Table 1).

**Table 1.** *Terpene contents in untreated leaves of* Nicotiana tabacum *‘Wisconsin’* Data are averages (±SE) of six independent replicates. nd, not detected.

Compound	Foliage terpene content (nmol g^−1^ DM)
Camphene	nd
Δ^3^-Carene	43 ± 7
Limonene	16 ± 5
(*E*)-β-Ocimene	nd
α-Pinene	20 ± 3
β-Pinene	10 ± 3
α-Caryophyllene	nd
α-Muurolene	nd

### Elicitation rate of emissions of LOX volatiles, mono- and sesquiterpenes

At the initial enhancement, hexanal emission was *ca* five times higher than pentanal emission upon ozone stress, but the hexanal elicitation rate was almost equal to the pentanal elicitation rate. Furthermore, at 48 h of recovery, the hexanal emission rate was three times higher than the pentanal emission rate, but its elicitation rate was two times lower than the pentanal elicitation rate (Fig. 4A–D). Ozone had a statistically significant impact (*P*<0.001) on the elicitation rates of hexanal and pentanal (Fig. 4C, D).

**Fig. 7.**
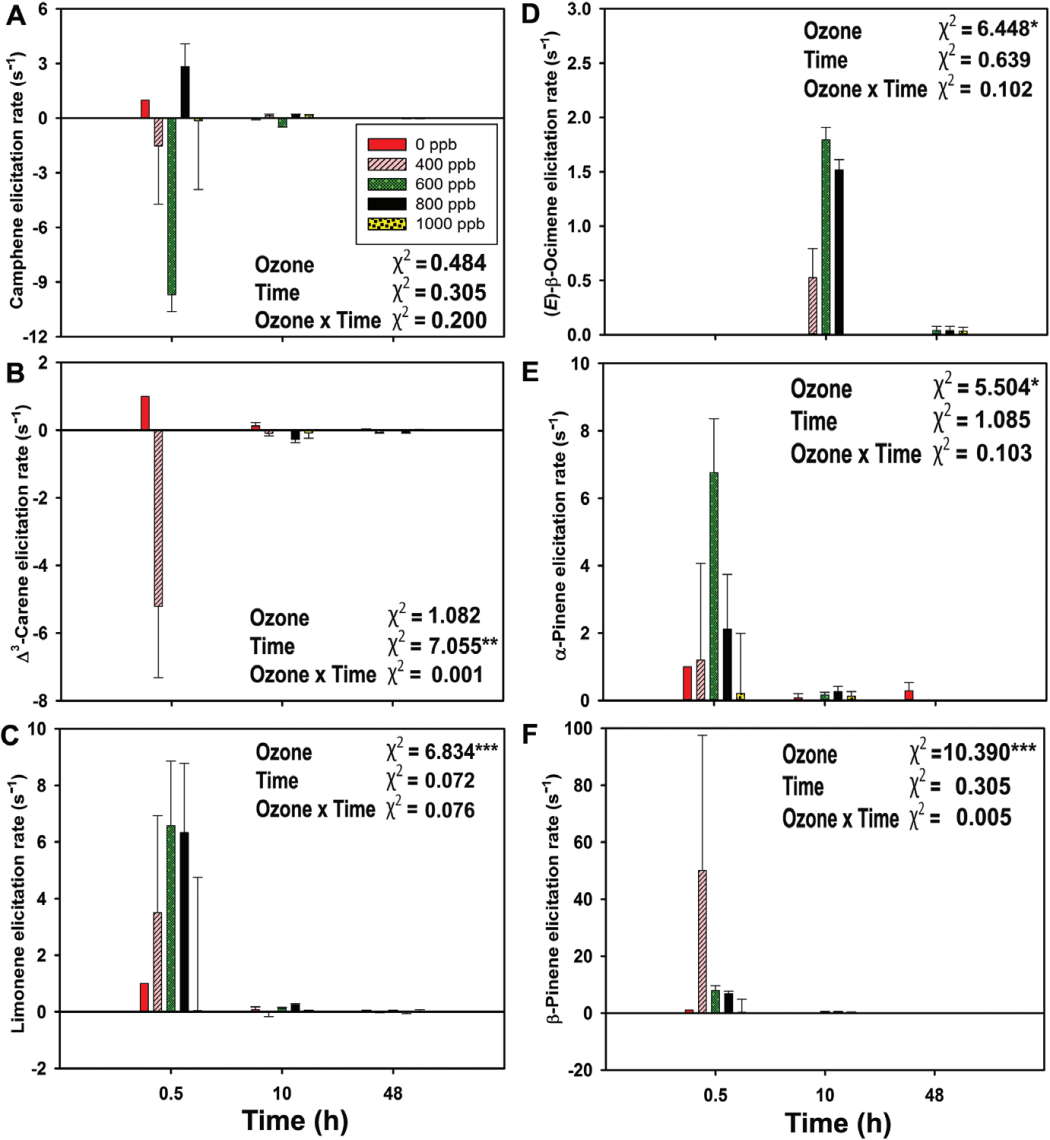
Average (+SE) elicitation rate of monoterpenes in mature leaves of 10- to 12-week-old *N. tabacum* ‘Wisconsin’ at 0.5, 10, and 48 h of recovery after ozone exposure at 0, 400, 600, 800, and 1000 ppb for 30 min in a custom-made cylindrical double-walled glass chamber at 25 °C. The elicitation rate of emissions is defined as the rate of change of emission (positive values indicate an increase of the rate and negative values a decrease of the rate). Data are averages of three independent replicates. Individual effects of ozone, recovery time (Time), and their interactions on emission rates were tested by GLM with maximum likelihood model fitting. Wald’s chi-square (χ^2^) test statistics and statistical significance are indicated as *P<0.05, **P<0.01, ***P<0.001. (This figure is available in color at *JXB* online.)

β-Pinene was the monoterpene elicited to the greatest degree, followed by limonene, α-pinene, and (*E*)-β-ocimene, although limonene was the highest emitted monoterpene (Fig. 7). The lowest elicitation rate was observed for camphene. The Δ^3^-carene emission rate was *ca* four times higher than the camphene emission rate but the elicitation rate of both compounds was almost equal (Figs 5 and 7). Ozone had a statistically significant impact on elicitation rates of limonene, (*E*)-β-ocimene, α-pinene, and β-pinene ((Fig. 7C–F)). The elicitation rate for (*E*)-β-ocimene was positive through the entire recovery period (Fig. 7D).

α-Muurolene was the most strongly elicited sesquiterpene, followed by α-caryophyllene. For both sesquiterpenes, ozone and recovery time significantly affected the elicitation rates (Fig. 6C, D). At 0.5 h of recovery, the α-muurolene elicitation rate was *ca* four times higher than the α-caryophyllene elicitation rate, although the emission rate of α-caryophyllene was *ca* two times higher than that of α-muurolene. At 48 h of recovery, the elicitation rate of both compounds reached the lowest level (Fig. 6A–D).

### Emissions of LOX volatiles, mono- and sesquiterpenes in relation to stomatal ozone uptake and non-stomatal ozone deposition rates

**Fig. 8.**
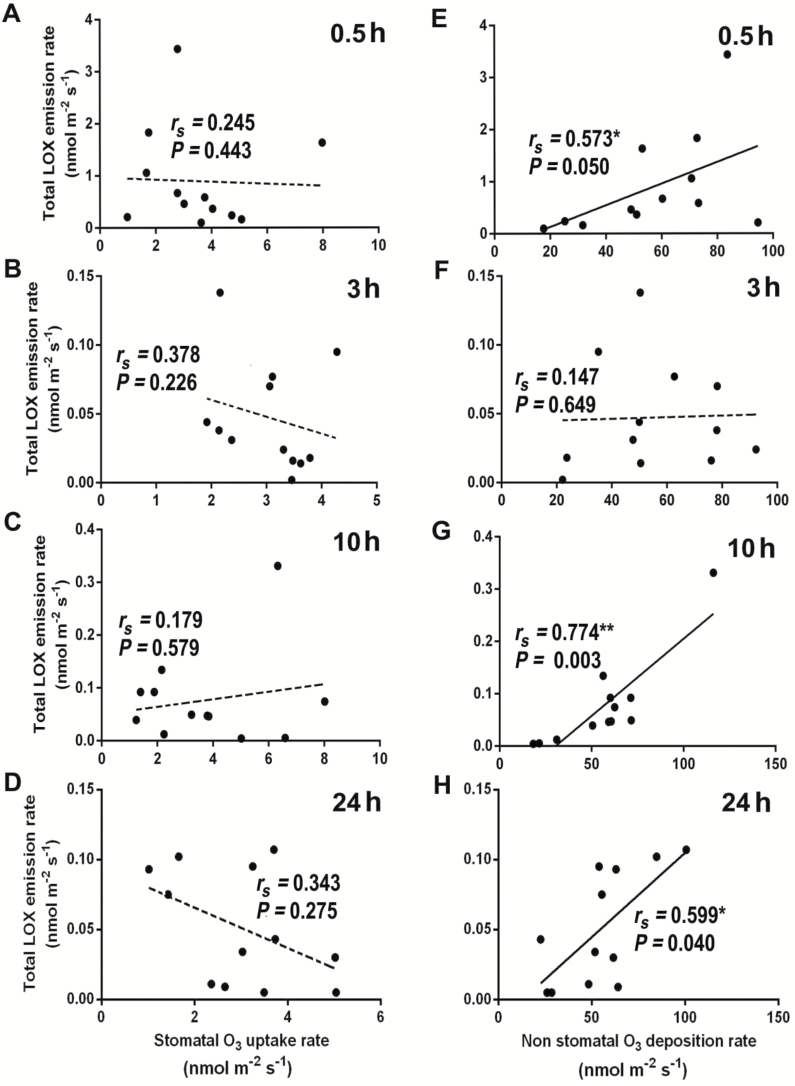
Correlations of total LOX volatile emission rates with stomatal ozone uptake rate (A–D) and non-stomatal ozone deposition rate (E–H) at different times (0.5, 3, 10, and 24 h) during recovery in mature leaves of 10- to 12-week-old *N. tabacum* ‘Wisconsin’. Each data point indicates an individual replicate. Data were fitted by Spearman correlation. Non-significant regressions (*P*>0.05) are shown by dashed lines. Statistical significance is indicated as *P<0.05, **P<0.01.

**Fig. 9.**
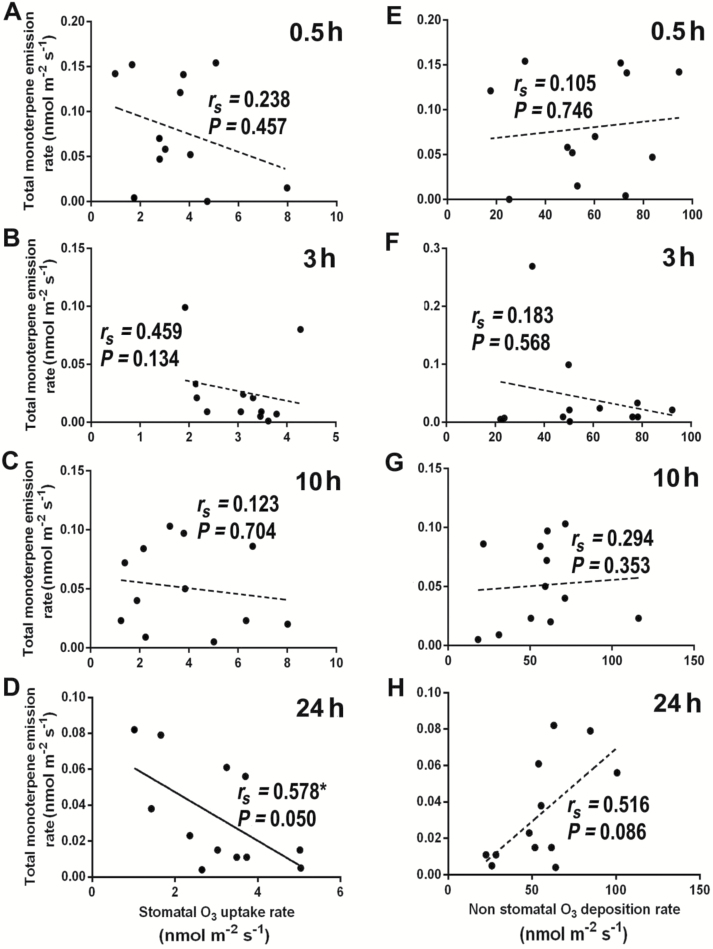
Correlations of total monoterpene emission rates with stomatal ozone uptake rate (A–D) and non-stomatal ozone deposition rate (E–H) at different times (0.5, 3, 10, and 24 h) through recovery in mature leaves of 10- to 12-week-old *N. tabacum* ‘Wisconsin’. Data were fitted by Spearman correlation. Non-significant regressions (*P*>0.05) are shown by dashed lines. Statistical significance is indicated as: *P<0.05.

**Fig. 10.**
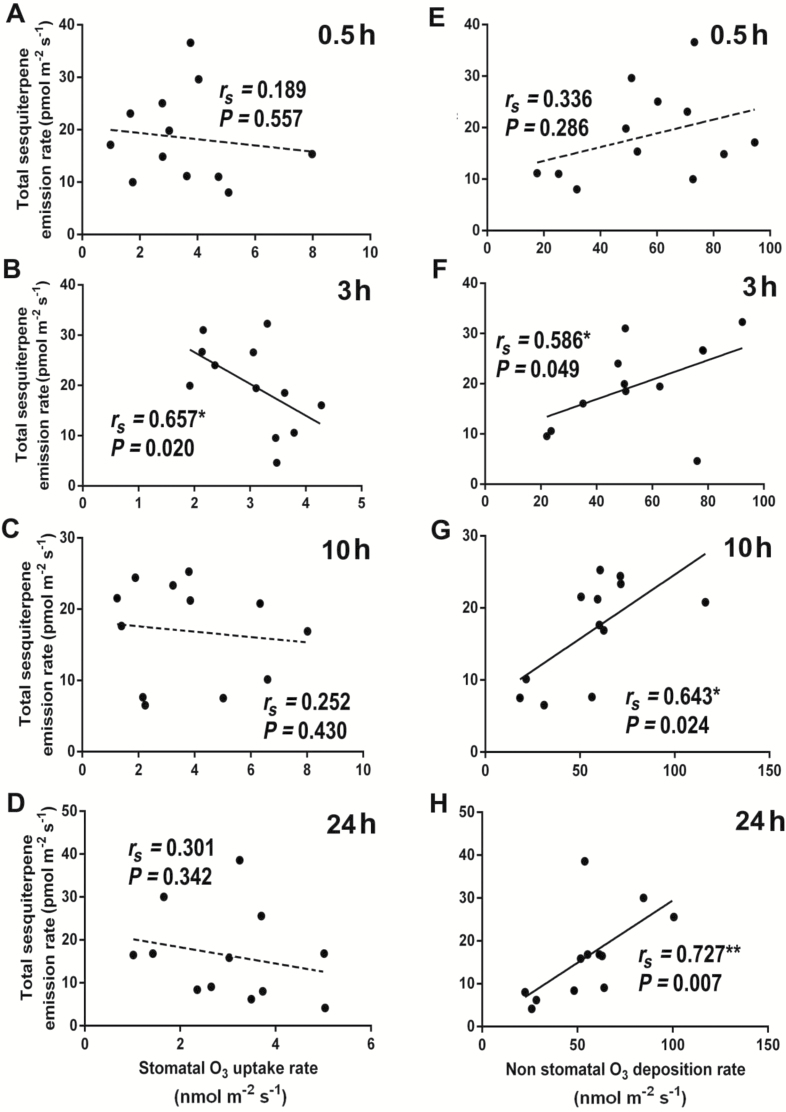
Correlations of total sesquiterpene emission rates with stomatal ozone uptake rate (A–D) and non-stomatal ozone deposition rate (E–H) at different times (0.5, 3, 10, and 24 h) during recovery in mature leaves of 10- to 12-week-old *N. tabacum* ‘Wisconsin’. Data were fitted by Spearman correlation. Non-significant regressions (*P*>0.05) are shown by dashed lines. Statistical significance is indicated as: *P<0.05, **P<0.01.

Overall, the total LOX volatile emission rate was poorly correlated with stomatal ozone uptake rate (Fig. 8A–D), but the correlations with non-stomatal ozone deposition rate (Fig. 8E–H) were positive and statistically significant at 0.5, 10, and 24 h since the exposure (Fig. 8E, G, H). In the case of total monoterpenes, a statistically significant negative correlation between stomatal ozone uptake rate (Fig. 9D), and a marginally significant positive correlation between non-stomatal ozone deposition rate (Fig. 9H) and monoterpene emission were observed at 24 h of recovery. For sesquiterpenes, the total emission rate was negatively correlated with stomatal ozone uptake rate at all recovery times (Fig. 10A–D), and positively and significantly correlated with ozone deposition rate at 3, 10, and 24 h of recovery (Fig. 10F, G, H).

## Discussion

### Changes in leaf photosynthetic characteristics upon acute ozone stress

Acute ozone concentrations caused a sharp decline in the maximum dark-adapted photosystem II quantum yield (*F*_v_/*F*_m_), especially at the exposures of 800 and 1000 ppb (Fig. 1). A reduction in *F*_v_/*F*_m_ is typically associated with sustained photoinhibition due to damage at PSII (Osmond *et al.*, 1999; Demmig-Adams and Adams, 2006). In fact, studies have demonstrated that exposure of leaves to elevated ozone results in increasingly deactivated PSII centres, thereby reducing the photochemical capacity of PSII (Guidi and Degl’Innocenti, 2008). Photoinhibitory effects of ozone are primarily caused by the overall net reduction of the D1 core protein of PSII (Adir *et al.*, 2003; Singh *et al.*, 2009).

There is also evidence that elevated ozone inhibits photosynthesis in a time-dependent manner due to suppression of the Calvin cycle (Guidi and Degl’Innocenti, 2008). In potato (*Solanum tuberosum*) leaves, elevated ozone inhibited the activity and reduced the quantity of ribulose-1,5-bisphosphate carboxylase/oxygenase leading to a reduced rate of photosynthesis (Dann and Pell, 1989) as was also observed in our study (Fig. 2A). However, the reductions in the photosynthetic rate and intercellular CO_2_ concentration were associated with a decline of stomatal conductance to water vapour (Fig. 2A–C). Although net assimilation rate and stomatal conductance typically decrease in parallel through abiotic stresses (Copolovici *et al.*, 2012; Kask *et al.*, 2016, Kanagendran *et al.*, 2018), this result indicates that the inhibitory effect of elevated ozone on net assimilation was at least partly associated with stomatal closure.

The reductions in net assimilation rate, stomatal conductance to water vapour, and intercellular CO_2_ concentration were strongly correlated with ozone concentration during the exposure (Figs 2A–C), indicating a dose-dependent response as observed in several studies (Guidi *et al.*, 2001; Degl’Innocenti *et al.*, 2002; Akhtar *et al.*, 2010). Despite treatment with high ozone concentrations, the recovery of photosynthetic characteristics at 48 h after ozone exposure indicates the high degree of ozone resistance of *N. tabacum* ‘Wisconsin’ used in our study (see Supplementary Fig. S1). However, from an ecological perspective, we note that the recovery times of 24–48 h are too long for a stress factor such as ozone that has a diurnal cycle. Another exposure cycle on the following day would not allow for such recovery to take place.

### Effect of acute ozone exposure on the emission of lipoxygenase pathway volatiles

Multiple lipoxygenases are constitutively active in plant cells, and once their substrates, polyunsaturated fatty acids, become available, LOX volatiles are rapidly released from membranes (Feussner and Wasternack, 2002). Typically, the release of polyunsaturated fatty acids from membranes and concomitant release of LOX volatiles are considered to be an acute stress response associated with major membrane-level damage (Jiang *et al.*, 2016, 2017). However, the situation with ozone might be more complicated as ozone itself can cause oxidative degradation of membrane lipids as well as oxidize cuticular lipids on the leaf surface (Senser *et al.*, 1987; Slimestad *et al.*, 1995).

The typical LOX volatiles emitted upon acute abiotic stress are (*E*)-2-hexenal, (*Z*)-3-hexenol, 1-hexanol, and (*Z*)-3-hexenyl acetate (Heiden *et al.*, 2003; Copolovici and Niinemets, 2010; Copolovici *et al.*, 2012). Indeed, Heiden *et al.* (1999) reported emission of these classical LOX volatiles upon acute ozone fumigation of 175 ppb for 5 h and 150 ppb for 11 h from ozone-sensitive *N. tabacum* ‘Bel W3’ and ozone-resistant *N. tabacum* ‘Bel B’. In our study, however, the emission of (*E*)-2-hexenal and (*Z*)-3-hexenol was observed at trace level at the highest ozone doses, and there was no emission of 1-hexanol and (*Z*)-3-hexenyl acetate upon elevated ozone. This suggests that the impact of ozone exposure on LOX volatiles depends on ozone concentration and exposure duration used and also varies among cultivars with different ozone sensitivity. In our study, we observed that the oxygenated volatile emissions were dominated by the saturated aldehyde hexanal followed by the saturated aldehyde pentanal and that these emissions were related to ozone concentration in a dose-dependent manner right after ozone exposure at 0.5 h (Figs 3A and 4A, B). The formation of hexanal is partly attributed to the breakdown of membrane lipids by lipoxygenase and hydroperoxide lyase enzymes (Heiden *et al.*, 2003), and thus the emission of hexanal following ozone fumigation may be associated with the direct effect of the lipoxygenase cleavage of fatty acids in the leaf membranes exposed to ozone (Paoletti, 2006). Similar to our study, Carriero *et al.* (2016) observed that the dominant LOX volatile was hexanal emitted following short-term acute ozone exposures in silver birch (*Betula pendula*). All these reports suggest that hexanal is a ubiquitous LOX volatile product emitted in relation to acute ozone stress. Nevertheless, we cannot rule out alternative biochemical reactions leading to saturated aldehyde formation (Wildt *et al.*, 2003) or direct reactions of ozone with leaf surface lipids or elicitation of LOX volatile production within leaf surface structures such as secretory cells feeding leaf glandular trichomes. At least the rapid cessation of LOX volatile emissions (Figs 3A and 4A, B) suggests that even the highest ozone exposures did not result in major cellular damage, consistent with almost full recovery of leaf photosynthetic characteristics (Figs 1 and 2 and Supplementary Fig. S1).

### Effect of acute ozone exposure on the emission of mono- and sesquiterpenes

Mainly due to the presence of glandular trichomes, *N. tabacum* is a constitutive low-level terpene emitter under non-stressed conditions. Relatively low levels of terpene emissions observed for non-stressed leaves are likely associated with a limited storage capacity for monoterpenes in glandular trichomes in tobacco (Table 1; Ohara *et al.*, 2003), compared with less volatile diterpene storage in trichomes (Cui *et al.*, 2011).

Upon ozone exposure, mono- and sesquiterpene emissions were generally enhanced, but the responses varied in time for different ozone doses (Figs 3B, C, 5, and 6A, B). Although total monoterpene emission was enhanced by ozone, it peaked at ozone concentrations of 400–600 ppb at different times during recovery and ultimately decreased to levels below that observed in control leaves at 48 h after exposure (Fig. 3B). In contrast, sesquiterpene emissions were more exposure dose dependent and weakly decreased over the recovery period (Fig. 3C). This difference between the two terpenoid classes is puzzling and might be associated with different metabolic pathways responsible for synthesis of these volatiles. In the case of monoterpenes, the synthesis occurs through the plastidial 2-C-methyl-D-erythritol 4-phosphate (MEP)–1-deoxy-D-xylulose 5-phosphate (DOXP) pathway, whereas sesquiterpene synthesis occurs through the mevalonate-dependent pathway in the cytosol (Rajabi Memari *et al.*, 2013; Rosenkranz and Schnitzler, 2013; Pazouki and Niinemets, 2016). In the mesophyll cells, the MEP–DOXP pathway activity is strongly dependent on immediate availability of NADPH and ATP generated by photosynthetic electron transport (Niinemets *et al.*, 2002; Rasulov *et al.*, 2009), and thus modifications in the activity of photosynthesis are expected to impact the substrate availability for monoterpene synthesis. In contrast, no such photosynthetic control is expected on the rate of sesquiterpene synthesis. Indeed, elevated ozone, 800–1000 ppb, significantly inhibited photosynthesis, and in turn it decreased emission rates of monoterpenes compared with the control treatment, suggesting that such a substrate-level control might be possible. However, monoterpene emission was actually initially enhanced for lower ozone concentrations and the substrate-level control also does not explain the decline of monoterpene emission, especially at 48 h when photosynthesis had almost fully recovered (cf. Figs 1, 2 and 3B, and Supplementary Fig. S1). Such a discrepancy might be associated with the circumstance that under ozone stress, a higher proportion of MEP–DOXP pathway products might have been allocated to the replacement of non-volatile larger isoprenoids, such as chlorophyll and carotenoids, oxidized upon ozone exposure (Pazouki *et al.*, 2016). In addition, as the stress might alter the allocation of photosynthates between carbon storage and biosynthesis of new chemicals (Fichtner *et al.*, 1994), full recovery of photosynthesis right after stress does not always guarantee that photosynthetic products will be high enough to support the rate of biosynthesis of secondary compounds.

An alternative explanation to the observed discrepancy between changes in photosynthesis and monoterpene emissions is that monoterpenes were primarily emitted from terpene pools stored in glandular trichomes (Table 1). Ozone exposure can enhance trichome emissions by damage to the trichome surface, thereby enhancing terpene permeance (Jud *et al.*, 2016). On the other hand, ozone itself can oxidize the terpenes released at a constant rate from trichomes (Pinto *et al.*, 2007), and this could explain the decreased emissions at the highest ozone concentrations (Fig. 3B). Furthermore, once damaged, discharge of terpene pools can be responsible for the monoterpene emission rates such that they reached values lower than those observed in control leaves. In the case of sesquiterpenes, changes in the emissions can also be explained by ozone effects on trichome surface permeability, albeit that sesquiterpenes are much less volatile than monoterpenes (Copolovici and Niinemets, 2005; Copolovici and Niinemets, 2015), and thus emptying the pools is expected to take much longer than for monoterpenes. However, this still does not explain why there was an apparent dose dependence for sesquiterpenes and not for monoterpenes.

### Contribution of terpenoid synthesized *de novo* and from storage pools to the terpenoid emission

Quantitative estimation of terpene contents in tobacco leaves demonstrated that Δ^3^-carene was the most abundant monoterpene stored in fresh leaf tissues followed by α-pinene, limonene, and β-pinene. However, the blend of monoterpenes emitted by both ozone-treated and untreated leaves was dominated by limonene, followed by α-pinene and β-pinene (Fig. 5). Therefore, a major portion of the emitted limonene is likely synthesized *de novo*, but camphene and (*E*)-β-ocimene were fully *de novo* synthesized in this study. Similarly, as there were no traces of α-caryophyllene and α-muurolene found in the leaf tissues, those two sesquiterpenes were ultimately *de novo* synthesized (Table 1 and Fig. 6A, B).

### Ozone-dependent modification of the blend of emitted terpenoids and time kinetics of elicitation rates of LOX volatiles and terpenoids

Several studies have demonstrated that upregulation of terminal enzymes following a stress was responsible for the enhanced emission of volatile isoprenoids (Singh and Johnson-Flanagan, 1998; Frank *et al.*, 2009; Pateraki and Kanellis, 2010). In a study with the ozone-sensitive tobacco cultivar ‘Bel W3’, Beauchamp *et al.* (2005) further demonstrated significant emissions of methyl salicylate (MeSA), suggesting a major physiological stress response upon ozone exposure in this sensitive cultivar. In our study, emissions of six monoterpenes and two sesquiterpenes were observed but no traces of MeSA. Lack of MeSA emission together with the lack of characteristic LOX volatiles (see above) further suggests that the observed modifications in terpenoid emissions, particularly Δ^3^-carene, α-pinene, and β-pinene, in this ozone-resistant cultivar primarily reflected changes in the constitutive emissions, most likely associated with changes in trichome surface permeability, further supported by correlation analysis and solvent extract analysis (Figs 8–10 and Table 1).

Nevertheless, lack of storage capacity and the characteristic emission response observed suggested elicitation of *de novo* synthesis of (*E*)-β-ocimene upon ozone exposure. In particular, relatively low-level emissions of (*E*)-β-ocimene were detected at later stages during recovery, with the emissions first observed at 3 h and peaking at 10 h since the exposure (Fig. 5D), suggesting that ozone exposure did activate defence responses. (*E*)-β-Ocimene has been considered an iconic stress monoterpene, and its emissions have been shown to be elicited upon biotic and abiotic stresses including exposure to high doses of ozone in *Phaseolus vulgaris* (Li *et al.*, 2017), cold and heat shock in *Solanum lycopersicum* (Copolovici *et al.*, 2012; Pazouki *et al.*, 2016), feeding by larvae of beet armyworm (*Spodoptera exigua*) in *Medicago truncatula* (Navia-Giné *et al.*, 2009), and feeding by larvae of common white wave (*Cabera pusaria*) in leaves of *Alnus glutinosa* (Copolovici *et al.*, 2011).

Sesquiterpenes, including α-caryophyllene, are also commonly considered stress-inducible (Lung *et al.*, 2016), but in our study it was α-muurolene, emission of which was lacking in control leaves and was induced during the recovery phase (Fig. 6B). Thus, this evidence suggests that ozone treatment could have changed the expression of several terpene synthase genes in our study, leading to a certain modification in the blend of emitted volatiles, albeit the influence of *de novo* gene expression on total emission was likely minor in our study.

A great variability in the emission rates of each compound, despite similar elicitation rates, can be explained by several factors governing the underlying mechanism, such as the following. (i) Ozone exposure can trigger an array of reactions and mechanisms varying from elicitation of gene expression to protein turnover and signal transduction, and all these process are time consuming. At the onset of ozone treatment, LOX volatile elicitation rate is primarily determined by the rate of free fatty acid break-down, resulting in an initial emission burst; higher emission rates of mono- and sesquiterpenes and concomitantly higher elicitation rate can further partly rely on the rapid increase of trichome permeability and, thus, the release of stored terpenes. On the other hand, the depletion of terpene contents in the storage structures leads to declining elicitation rates, ultimately even to negative values. (ii) Expression of an array of genes in the LOX, MEP, and MVA pathways is elicited after some hours of stress application and has a substantial impact on emission responses along with substrate level control (Pazouki *et al.*, 2016), especially at 10 h of elicitation in this study. However, at 48 h of recovery, the elicitation rate became negative for most of the compounds, reflecting lower emission rates than in controls. And (iii) there are physiological and physico-chemical limitations within the leaf mesophyll that control the volatile diffusion at the leaf–atmosphere interface, such as the availability of ATP, NADPH, and glyceraldehyde 3-phosphate; maximum activity of rate-controlling enzymes; compound gas-phase partial pressure; and aqueous- and lipid-phase concentrations (Niinemets and Reichstein, 2003 for a review). While physiological limitations can affect the maximum synthesis rate of volatiles, physico-chemical limitations on *de novo* synthesized volatiles, except for lipophilic compounds such as non-oxygenated terpenes, can have a substantial influence on the time lags between synthesis and emission.

### Emission responses of stress volatiles in response to stomatal ozone uptake and non-stomatal ozone deposition

Ozone uptake by the leaves from the ambient atmosphere occurs by two means, stomatal uptake and by surface deposition. These means of uptake have fundamentally different physiological impacts. In addition, once taken up, the ultimate damaging effect of ozone depends on the extent to which ozone is scavenged by water-soluble antioxidants within the aqueous phase in cell walls and by volatile antioxidants within the leaf gas phase as well as by lipid-soluble volatile and non-volatile antioxidants. Among these volatile antioxidants (Kurpius and Goldstein, 2003; Goldstein *et al.*, 2004; Bouvier-Brown *et al.*, 2009), mono- and sesquiterpenes are particularly reactive with ozone (Fares *et al.*, 2010*a*).

Analysis of the distribution of ozone flux between stomatal uptake and surface deposition indicated that stomatal absorption of ozone was much lower than the non-stomatal deposition for *N. tabacum* ‘Wisconsin’ (Figs 8–10). We argue that the high surface deposition flux reflects the presence of terpene-filled glandular trichomes on the leaf surface, and accordingly direct reaction of ozone with unsaturated terpenoids, e.g. unsaturated semi-volatile compounds such as *cis*-abienol (C_20_H_34_O), present on the *N. tabacum* leaf surface that can act as an efficient sink for ozone and a powerful chemical barrier against stomatal ozone uptake at the leaf surface (Jud *et al.*, 2016)—but, of course, other terpenoids including mono- and sesquiterpenes can contribute to ozone detoxification. Furthermore, non-stomatal deposition, itself due to volatiles produced by the plant, not only occurs at the leaf surface, but also can occur within the leaf boundary layer and even outside the boundary layer (Altimir *et al.*, 2006; Fares *et al.*, 2008).

Comparisons of the correlations of total emission rates of LOX volatiles, mono- and sesquiterpenes with stomatal ozone uptake and with non-stomatal ozone deposition indicated that the correlations were stronger with non-stomatal deposition (Figs 8A–D, 9A–D, and 10A–D*vs*. Figs 8E–H, 9E–H, and 10E–H). Counterintuitively, in several cases, total emission rates of mono- and sesquiterpenes were negatively correlated with stomatal ozone uptake. There can be several explanations for these negative correlations. First, when ozone enters the leaf mesophyll cells it can rapidly react with water-soluble antioxidants such as putrescine and apoplastic ascorbate, and therefore the amount of ozone trigging stress is typically much lower than that entering through stomata (Laisk *et al.*, 1989). Second, volatile isoprenoids can react directly with ozone inside the leaves or with ozone-induced reactive oxygen species (ROS; Loreto and Fares, 2007), resulting in lower rates of emission of mono- and sesquiterpenes than their biosynthesis rates. Lack of correlation immediately after the exposure suggests that terpene reactions with ozone inside the leaves were unlikely to have played a role in these negative correlations. Yet, there is typically a secondary rise of ROS several hours after the initial stress impact (Beauchamp *et al.*, 2005; Jiang *et al.*, 2017; Li *et al.*, 2017), and it is plausible that the correlations observed 3–24 h after the ozone exposure reflected isoprenoid reactions with the endogenously generated ROS.

The positive correlations among non-stomatal ozone deposition and total LOX volatile and mono- and sesquiterpene emissions (Figs 8E–H, 9E–H, and 10E–H) are wholly consistent with the hypothesis of the surface release of volatiles from glandular trichomes or impacted secretory cells. These results collectively suggest that a significant part of volatile emission in response to ozone exposure was release from the leaf surface.

## Conclusions

Ozone is believed to affect plants in a dose-dependent manner, i.e. the effects depend both on ozone concentration above the threshold value (concentration eliciting a physiological response, typically a low value of 40 ppb is assumed) and the duration of the ozone exposure (Beauchamp *et al.*, 2005; Niinemets *et al.*, 2010), but as our study demonstrates, the ozone dose responses can be complicated due to surface reactions. Taken together, these results from one stress cycle, with a long time for recovery, indicate that acute ozone exposure led to a significant reduction of foliar gas-exchange characteristics, but the reductions in all these characteristics were almost fully reversible even at the highest ozone concentrations underscoring the high ozone resistance of *N. tabacum* ‘Wisconsin’. The emission rate of LOX volatiles was dose dependent, whereas the dose dependences for terpenes, especially for monoterpenes, were weaker, but *de novo* elicitation of terpenoid synthesis was moderate, again corroborating the high ozone resistance of this genotype.

In this study, most of the ozone flux to the leaf was due to non-stomatal ozone deposition, and non-stomatal ozone deposition scaled with the release of terpenes, suggesting that terpenoid emission responses to ozone mostly reflected modification of terpene release from trichomes on the leaf surface. The results further suggest that the surprisingly high ozone resistance of *N. tabacum* ‘Wisconsin’ is likely due to surface reactions of volatiles released upon ozone exposure. This study demonstrated that, counterintuitively, in this ozone-resistant tobacco cultivar, the quantity of ozone uptake through stomata is not proportional to the volatile emission rates. Overall, there was a substantial variability of emission rates of each compound upon similar ozone uptake, suggesting that several genetic, physiological, and physiochemical factors control elicitation and emission of volatile compounds upon acute ozone stress in a complex manner. Further studies are required to understand the complex linkages between elicitation and emission due to genetic, physiological, and physiochemical factors in multiple tobacco cultivars with varying ozone surface and stomatal deposition fluxes.

## Supplementary data

Supplementary material is available at JXB online.

Fig. S1. Changes in leaf net assimilation rate (*A*), stomatal conductance to water vapour (*g*_s_), and intercellular CO_2_ concentration (*C*_i_) of mature leaves of 10- to 12-week-old *N. tabacum* ‘Wisconsin’ in relation to ozone concentration during exposure.

Supplementary Figure S1

## Abbreviations:

BVOC: biogenic volatile organic compound;
DOXP: 1-deoxy-D-xylulose 5-phosphate
GLM: generalized linear model
GLV: green leaf volatiles
LOX: lipoxygenase pathway
MEP: 2-C-methyl-D-erythritol 4-phosphate
MVA: mevalonate.
