# Supplementary material for: Ozone-triggered surface uptake and stress volatile emissions in Nicotiana tabacum ‘Wisconsin’
Source: J Exp Bot. 2017 Dec 30;69(3):681–97. doi: 10.1093/jxb/erx431 (PMC5853501; doi:10.1093/jxb/erx431)
Supplement: Supplementary Figure S1 [file erx431_suppl_supplementary_figure_s1.pdf]

MS title: Stress volatile emissions upon acute ozone exposure scale with foliage surface ozone uptake in ozone-resistant *Nicotiana tabacum* ‘Wisconsin’

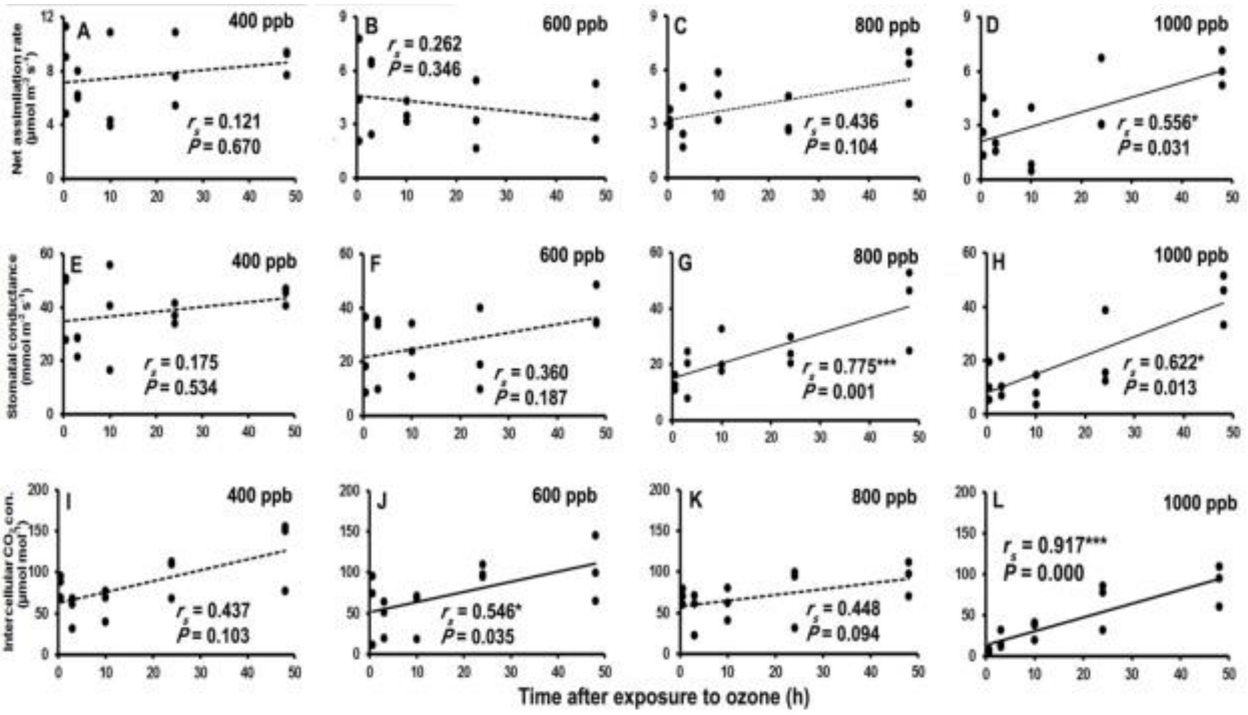

Supplementary Fig. S1. Changes in (A-D) leaf net assimilation rate (*A*), (E-H) stomatal conductance to water vapour (g_s), and (I-L) intercellular CO_2 concentration (C_i) of mature leaves of 10-12 weeks old *N. tabacum* ‘Wisconsin’ in relation to ozone concentration during exposure. The measurements were taken at 0.5, 3, 10, 24, and 48 h after ozone exposure at 0, 400, 600, 800, and 1000 ppb for 30 min in a custom-made cylindrical double-walled glass chamber 25 °C. All measurements were replicated at least thrice. Gas exchange measurements were conducted at leaf temperature of 25 °C, quantum flux density of 700 μmol m^{-2} s^{-1} at leaf surface, ambient CO_2 concentration of 380-400 μmol mol^{-1}, and relative humidity of 50–60%. Each data point corresponds to an individual replicate measurement. Data were fitted by Spearman correlations. Statistical significance of regressions is indicated as: * -P < 0.05, *** -P < 0.001.
